# Regulating Dendrite‐Free Zinc Deposition by Red Phosphorous‐Derived Artificial Protective Layer for Zinc Metal Batteries

**DOI:** 10.1002/advs.202200155

**Published:** 2022-04-24

**Authors:** Tian Wang, Qiao Xi, Yifan Li, Hao Fu, Yongbin Hua, Edugulla Girija Shankar, Ashok Kumar Kakarla, Jae Su Yu

**Affiliations:** ^1^ Department of Electronics and Information Convergence Engineering Institute for Wearable Convergence Electronics Kyung Hee University Yongin‐si Gyeonggi‐do 17104 Republic of Korea; ^2^ Frontiers Science Center for Flexible Electronics (FSCFE) Shaanxi Institute of Flexible Electronics (SIFE) Northwestern Polytechnical University 127 West Youyi Road Xi'an 710072 China; ^3^ Department of Physics Dongguk University Seoul 04620 Republic of Korea

**Keywords:** aqueous Zn‐ion batteries, artificial protective layer, dendrite‐free Zn deposition, Zn anode

## Abstract

Rational architecture design of the artificial protective layer on the zinc (Zn) anode surface is a promising strategy to achieve uniform Zn deposition and inhibit the uncontrolled growth of Zn dendrites. Herein, a red phosphorous‐derived artificial protective layer combined with a conductive N‐doped carbon framework is designed to achieve dendrite‐free Zn deposition. The Zn–phosphorus (ZnP) solid solution alloy artificial protective layer is formed during Zn plating. Meanwhile, the dynamic evolution mechanism of the ZnP on the Zn anode is successfully revealed. The concentration gradient of the electrolyte on the electrode surface can be redistributed by this protective layer, thereby achieving a uniform Zn‐ion flux. The fabricated Zn symmetrical battery delivers a dendrite‐free plating/stripping for 1100 h at the current density of 2.0 mA cm^–2^. Furthermore, aqueous Zn//MnO_2_ full cell exhibits a reversible capacity of 200 mAh g^–1^ after 350 cycles at 1.0 A g^–1^. This study suggests an effective solution for the suppression of Zn dendrites in Zn metal batteries, which is expected to provide a deep insight into the design of high‐performance rechargeable aqueous Zn‐ion batteries.

## Graphical abstract

An artificial protective layer based on zinc‐phosphorus (ZnP) alloying reaction is designed to regulate the deposition behavior of Zn ions. The synergistic effect of the ZnP alloy artificial protective layer and carbon framework enables a uniform electric field strength distribution and a homogeneous Zn‐ion flux on the electrode surface, which results in the dendrite‐free Zn plating/stripping.

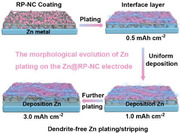

## 1 Introduction

Metallic zinc (Zn) with a high theoretical capacity (820 mAh g^–1^), low redox potential (‐0.762 V vs standard hydrogen electrode), and high abundance makes rechargeable aqueous Zn‐based batteries most promising intrinsically safe for large‐scale energy storage devices.^[^ ^1^ ^]^ Unfortunately, the passivation layer produced between the Zn metal and the electrolyte interface is different from the solid electrolyte interface (SEI) on the lithium metal surface, due to its instability, nonconducting of Zn ions, and high thermodynamic activity.^[^ ^2^ ^]^ During the continuous plating/stripping process, a heterogeneous distribution of Zn ions on the anode surface induces the growth of dendrites.^[^ ^3^ ^]^ The uncontrollable Zn dendrites will increase the Zn metal corrosion by electrolytes and greatly reduce the Coulombic efficiency (CE). Seriously, it will pierce the separator of batteries to cause internal shorting failure.^[^ ^4^ ^]^ Meanwhile, the side reactions (corrosion and hydrogen evolution) between the Zn anode and electrolyte will cause battery swelling, thus limiting the cycle life.^[^ ^5^ ^]^ Therefore, regulating the Zn electroplating behavior to achieve a uniform Zn deposition and restraining undesired side reactions will be of great value for enhancing the electrochemical performance of aqueous Zn‐ion batteries (AZIBs).

**Figure 1:**
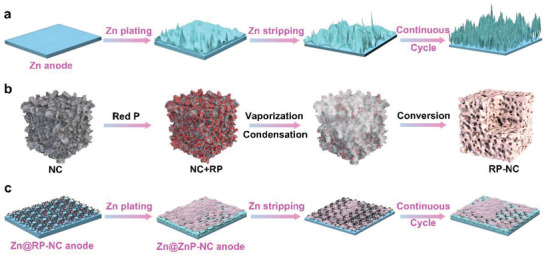
Schematic of the a) Zn stripping/plating process on pristine Zn anode, b) formation of the RP‐NC, and c) Zn stripping/plating process on Zn@RP‐NC anode.

According to the metal deposition model, the final morphologies of electroplated metals are largely affected by the distribution of metal ions at the electrode/electrolyte interface.^[^ ^6^ ^]^ Therefore, the uniform ion flux at the electrode/electrolyte interface is the key factor to control the deposition morphology.^[^ ^3b^ ^]^ Nonuniform Zn‐ion flux will lead to inhomogeneous nucleation, which triggers Zn dendrite formation and growth during the continuous plating/stripping processes (**Figure** **1**a).^[^ ^1^, ^7^ ^]^ To conquer the urgent issue, constructing an ideal SEI film onto Zn anode could be a promising strategy. Nevertheless, the intrinsic electrochemical stability of the Zn salt anions in the solution cannot form a Zn‐ion conducting SEI.^[^ ^8^ ^]^ Thus, artificial protective layers on Zn anode, as an alternative, have been proved to be an effective strategy.^[^ ^2^, ^3^, ^4^, ^9^ ^]^ These strategies can be divided into two classes, including the construction of the Zn host material and the establishment of the Zn/electrolyte interface, which depends on the Zn electrodeposited on the host material or the surface of the anode during the operation. However, most coating layers prepared by ex situ technology suffer from inhomogeneity, which may cause the protective layer to crack during continuous Zn plating/stripping.^[^ ^2^, ^9^ ^]^ In contrast, the protective layer obtained by chemical or electrochemical reactions usually exhibits a more uniform and stable structure, which makes it easier to achieve a uniform Zn‐ion flux.

In this work, a homogeneous artificial protective layer on Zn anode was designed to achieve dendrite‐free Zn deposition and promote the electrochemical performance of AZIBs. A dense and smooth Zn–phosphorus (ZnP) solid solution alloy artificial protective layer was formed by the in situ Zn plating. In this architecture, the excellent conductivity of the N‐doped carbon framework (NC) could regulate the uniform electric field distribution on the electrode surface. The ZnP‐NC protective layer can reshape the Zn‐ion flux near the interphase, resulting in Zn‐ion diffusion evenly on the Zn electrode surface. Therefore, the Zn anode equipped with ZnP alloy artificial protective layer and NC (Zn@ZnP‐NC) represented a dendrite‐free plating/stripping behavior over long time and a small voltage polarization in symmetrical batteries. In addition, the Zn@ZnP‐NC//MnO_2_ aqueous full cell delivered better electrochemical performance than the Zn//MnO_2_ full cell, indicating its great practical application prospects.

## 2 Results and Discussion

The fabrication process of ZnP‐NC artificial protective layer via plating Zn on the coating layer to obtain dendrite‐free Zn plating/stripping was summarized schematically in Figure 1b,c. Red phosphorus nanodots were anchored on the NC (RP‐NC) by the vaporization–condensation–conversion process. Subsequently, RP‐NC materials were evenly coated on the surface of the Zn foil (Zn@RP‐NC). Finally, the ZnP alloy artificial protective interlayer was obtained by in situ electrodeposition method (Zn@ZnP‐NC) to achieve the dendrite‐free deposition. The scanning electron microscope (SEM) images of Figure S1 (Supporting Information) revealed that the NC presented a 3D structure, which could effectively balance the current density and postpone the volume change during the metal plating/stripping, maintaining the structure stability of the electrode.^[^ ^10^ ^]^ Figure S2 (Supporting Information) displays the SEM image of the RP‐NC. After the RP was anchored on the NC, the structure of the NC remains intact.

**Figure 2:**
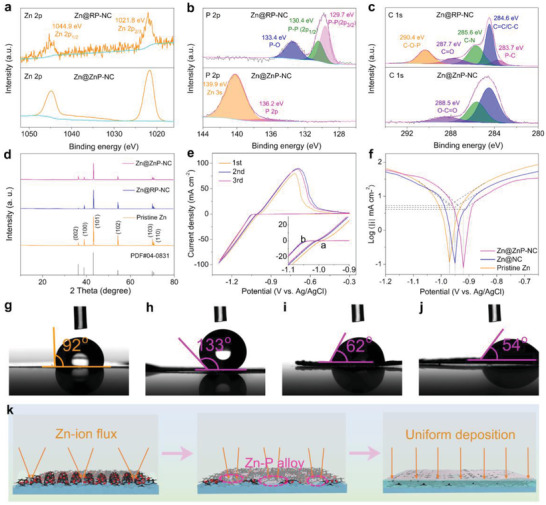
High‐resolution XPS spectra of a) Zn 2p, b) P 2p, and c) C 1s for the RP‐NC and ZnP‐NC (0.25 mA cm^–2^, 1.0 mAh cm^–2^) coating. d) XRD patterns. e) CV curves of Zn plating/striping on RP‐NC coated Ti foil. f) Linear polarization curves of the different electrodes. Contact angles of g) pristine Zn, h) Zn@RP‐NC, and Zn@ZnP‐NC with the capacities of i) 0.5 and j)1.0 mAh cm^–2^ at a current density of 0.25 mA cm^–2^. k) Schematic illustration of the evolution process of ZnP alloy layer.

The composition of the ZnP alloy artificial protective layer was characterized by X‐ray photoelectron spectroscopy (XPS) after plating Zn at 0.25 mA cm^–2^ with a capacity of 1.0 mAh cm^–2^ on the Zn@RP‐NC sample. The high‐resolution XPS Zn 2p spectra of both Zn@RP‐NC and Zn@ZnP‐NC displayed two distinct packs at 1044.9 and 1021.7 eV, which are attributed to Zn 2p_1/2_ and Zn 2p_3/2_, respectively (**Figure** **2**a).^[^ ^11^ ^]^ In detail, the P 2p spectrum of Zn@RP‐NC was fitted by three peaks which are related to P—P 2p_3/2_ (129.7 eV), P—P 2p_1/2_ (130.4 eV), and P—O (133.4 eV), respectively.^[^ ^12^ ^]^ However, the P 2p spectrum of Zn@ZnP‐NC was different from that of Zn@RP‐NC, the characteristic peak of P 2p moved to 135.6 eV, and a new peak of Zn 3s appeared at 139.9 eV, which might be attributed to the P atom entering into the Zn lattice (Figure 2b).^[^ ^13^ ^]^ The comparison of the C 1s spectrum is shown in Figure 2c. For Zn@RP‐NC, the peaks at 284.6, 285.6, and 287.6 eV can be attributed to the C═C/C—C, C—N, and C═O, respectively, whereas the peaks at 283.7 and 290.4 eV are assigned to the P—C and P—O—C bonds, respectively.^[^ ^14^ ^]^ The RP nanoparticles were bounded with NC by P‐C chemical bond, which will provide an excellent electrical contact for Zn deposition and achieve a uniform electric field distribution. Meanwhile, the disappearance of the P—C and P—O—C bonds was accompanied by the formation of O—C═O bonds at 288.5 eV in the C 1s spectrum of the Zn@ZnP‐NC,^[^ ^15^ ^]^ which might be related to the formation of ZnP chemical bonds. The N 1s XPS spectrum showed three different nitrogen species, including pyridinic‐N at 398.2 eV, pyrrolic‐N at 399.1 eV, and graphitic‐N at 400.8 eV as shown in Figure S3 of the Supporting Information.^[^ ^16^ ^]^ From the X‐ray diffraction (XRD) pattern, the peaks of pristine Zn correspond to (002), (100), (101), (102), (103), and (110) which are assigned by the PDF#04‐0831.^[^ ^4^, ^17^ ^]^ Furthermore, the same diffraction peaks of Zn@ZnP‐NC imply that Zn and RP form a solid solution alloy (Figure 2d).^[^ ^13^ ^]^

In consideration of the successful construction of the ZnP‐NC artificial protective layer, the nucleation growth behavior of Zn ions was investigated by the cyclic voltammetry (CV) curves for Ti@RP‐NC (Figure 2e), Ti@NC (Figure S4a of Supporting Information), and pristine Ti foil (Figure S4b of Supporting Information) as the substrate, respectively. In the first curves, the higher nucleation overpotential (NOP) of 58 mV was induced for the Ti@RP‐NC compared with the Ti@NC (37 mV) and pristine Ti (31 mV). The high NOP means that the PR‐NC coating layer reduces the deposition kinetics of Zn ions, which is beneficial to refine the deposited particles and achieve the uniform metal deposition.^[^ ^9^, ^18^ ^]^ The corrosion resistance was further studied through the linear polarization curves (Figure 2f). Compared with the pristine Zn, the corrosion potential of the Zn@NC and Zn@ZnP‐NC electrodes increased from ‐0.97 to ‐0.95 and ‐0.92 V, respectively. Additionally, the corrosion current density can also reflect the self‐corrosion reaction rate of the electrode. Here, the values of the Zn@ZnP‐NC and Zn@NC electrodes were 4.18 and 4.73 mA cm^–2^ respectively, which were lower than the pristine Zn electrode (5.50 mA cm^–2^). The higher corrosion‐positive potential and lower corrosion current of the Zn@ZnP‐NC electrode indicate that the anodic dissolution and hydrogen evolution reaction are effectively suppressed by this artificial protective layer.^[^ ^4^, ^19^ ^]^ Moreover, the electrochemical impedance spectroscopy (EIS) plots in symmetric batteries (Figure S5 of Supporting Information) revealed that the interfacial charge‐transfer resistance was reduced from 1043 Ω (pristine Zn) to 134 Ω (Zn@ZnP‐NC), indicating that the introduction of ZnP alloy artificial protective layer can produce faster charge transfer. Additionally, the ionic conductivity of ZnP‐NC protective layer was characterized by EIS plot. As shown in Figure S6 of the Supporting Information, the ZnP‐NC protective layer displays an ionic conductivity of 1.207 × 10^–4^ S cm^–1^, which indicates that the protective layer is conducive to the diffusion of Zn ions.^[^ ^3^, ^9^ ^]^

The wettability of the electrode surface plays an important role in the deposition behavior of Zn ions. The good wettability of the electrode surface can effectively improve the uniform distribution of Zn ions at the interface.^[^ ^4^, ^9^ ^]^ As shown in Figure 2g,h, the contact angle of the Zn@RP‐NC (133°) was significantly increased compared to the pristine Zn foil (pristine Zn) (92°), indicating that the RP‐NC coating layer was much more hydrophobic.^[^ ^20^ ^]^ Furthermore, the wettability properties of Zn@ZnP‐NC with different capacities are shown in Figure 2i (0.25 mA cm^–2^, 0.5 mAh cm^–2^) and Figure 2j (0.25 mA cm^–2^, 1.0 mAh cm^–2^). The contact angle of the Zn@ZnP‐NC with a capacity of 1.0 mAh cm^–2^ (54°) was smaller than that of the Zn@ZnP‐NC with a capacity of 0.5 mAh cm^–2^ (62°), which indicated that the formed ZnP alloy interface can effectively improve the hydrophilicity of the electrode. As a consequence, electrolyte ions can easily contact the metal anode, which is conducive to regulating the Zn‐ion flux, achieving a uniform Zn deposition.^[^ ^9d^ ^]^ In addition, variation of current versus time under an overpotential of ‐150 mV was performed to characterize the surface diffusion process of Zn ion.^[^ ^21^ ^]^ As shown in Figure S7 (Supporting Information), the current density of pristine Zn kept increasing over 400 s, which indicated a continuous two‐dimensional (2D) diffusion of Zn ion in the deposition progress.^[^ ^3b^ ^]^ In contrast, the current distribution of the Zn@RP‐NC electrode had a rapid decrease in the initial stage and was gradually stabilized after 100 s, which implied that the Zn@RP‐NC electrode had an initial 2D diffusion and followed by a continuous 3D diffusion.^[^ ^21b^ ^]^ This phenomenon indicates that the ZnP alloy layer is formed on the electrode surface at the initial stage of deposition. Meanwhile, the lower current density of the Zn@RP‐NC electrode indicates a uniform Zn deposition process without significant surface area increase, further confirming the dendrite‐free Zn deposition.^[^ ^19a^ ^]^ Hence, the schematic Zn deposition for Zn@RP‐NC can be described in Figure 2k. In the initial electrochemical deposition stage, a large contact angle causes a steep concentration gradient of the electrolyte on the electrode surface, which promotes the partial formation of ZnP alloy on the electrode surface. In the subsequent process, benefiting from the excellent wettability of the ZnP alloy, the ion concentration gradient in the electrolyte is gradually stabilized, achieving the uniform Zn‐ion flux, which is a prerequisite for dendrite‐free Zn deposition. In contrast, the weak wettability of the pristine Zn and the inherently uneven surface leads to less nucleation sites and aggravates the electrolyte concentration gradient. The subsequent process exhibits uneven deposition and dendrite growth. Figure S8 (Supporting Information) displays the corresponding schematic Zn deposition for pristine Zn.

To further validate the positive effect of the ZnP‐NC artificial protective layer on the uniform Zn deposition, the evolutions of Zn deposition morphologies of the pristine Zn, Zn@NC, and Zn@RP‐NC electrodes were observed by the SEM images. The rough surface (Figure S9a,b of Supporting Information, 0.25 mA cm^–2^, 0.5 mAh cm^–2^) indicated inhomogeneous Zn deposition caused by the steep concentration gradient on the Zn electrode. Meanwhile, many pores and cracks on the Zn electrode induced uneven Zn‐ion flux. Highly polarized deposition continues until the battery failure.^[^ ^1^, ^22^ ^]^ When 0.5 mAh cm^–2^ Zn was plated on the Zn@NC current collector, the vertically oriented Zn platelets were observed on the NC (Figure S10a,b of Supporting Information), which was attributed to the excellent conductivity of the NC to achieve the rapid Zn deposition. Unfortunately, the irregular Zn platelets on the outer surface will continue to grow into Zn dendrites, making it difficult to achieve long‐term Zn deposition.^[^ ^1^, ^11^, ^23^ ^]^ In contrast, a relatively flat and dense plated layer was observed when plating 0.5 mAh cm^–2^ Zn on the Zn@RP‐NC electrode surface, as shown in Figure S11a of Supporting Information. It can be found from the cross‐sectional SEM image that Zn ions are preferentially deposited on the surface and form a dense protective layer (Figure S11b of Supporting Information). As shown in Figure 2k, this phenomenon is depicted by the schematic of the Zn deposition.

**Figure 3:**
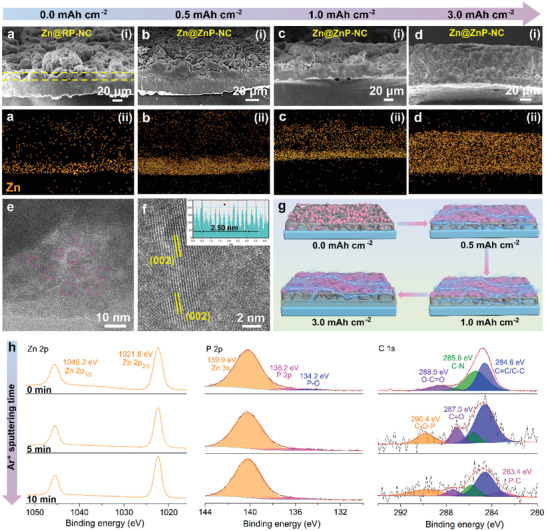
Cross‐sectional SEM images and their corresponding EDS mapping images of a‐i,ii) the Zn@RP‐NC and the Zn@RP‐NC anode deposited Zn at 0.25 mA cm^–2^ with different deposition capacities of b‐i,ii) 0.5 mAh cm^–2^, c‐i,ii) 1.0 mAh cm^–2^, and d‐i,ii) 3.0 mAh cm^–2^, respectively. e,f) HRTEM images of the ZnP alloy and NC. g) Schematic illustration of morphology evolution under quantitative Zn deposition. h) High‐resolution XPS spectra of the Zn 2p, P 2p, and C 1s for the coating layer depending on the Ar^+^ sputtering time.

Furthermore, the morphological evolution of Zn plating on the Zn@RP‐NC electrode was studied by ex situ cross‐sectional SEM images and corresponding energy‐dispersive X‐ray spectroscopy (EDS) mappings with different deposition capacities. **Figure** **3**a‐i shows the cross‐sectional SEM image of the fresh Zn@RP‐NC electrode. A coating layer with a thickness of 10–15 µm can be observed, which served as a contrast for the subsequent plating of the morphological evolution. As shown in Figure 3a‐ii and Figure S12a‐i–iii of Supporting Information, the EDS mappings displayed the distribution of the Zn, P, C, and N elements, and there is no obvious intersection between the Zn and other elements. When 0.5 mAh cm^–2^ Zn was plated on the Zn@RP‐NC electrode (Figure 3b‐i), even though the thickness of the Zn substrate remained unchanged, there was obvious Zn element accumulation on the surface of the electrode (Figure 3b‐ii). Combined with the distribution of P, C, and N element mapping images (Figure S12b‐i–iii of Supporting Information), it could be inferred that the deposited Zn ions form ZnP alloy on the electrode surface. When increasing the Zn deposition capacity to 1.0 mAh cm^–2^, the SEM image revealed a denser deposition morphology without dendrites (Figure 3c‐i). As shown in Figure 3c‐ii, the Zn element was uniformly distributed on the electrode surface. Meanwhile, the thickness of the Zn substrate increased, which implied that the Zn ions were deposited on the substrate. Under a higher deposition capacity of 3.0 mAh cm^–2^, the plated Zn displayed a much denser morphology (Figure 3d‐i). Combined with the corresponding element mapping images (Figure 3d‐ii and Figure S12d‐i–iii of Supporting Information) and cross‐sectional SEM images (Figure S13a,b of Supporting Information), it could be found that the electrode structure remained intact even though the electrode thickness increases apporoximately two times. The above phenomenon confirms that the ZnP alloy layer can regulate the uniform Zn‐ion flux and thus, the dendrite‐free Zn depositions are easily achieved.

To further emphasize the existence of ZnP alloy phase, the interlayer (0.25 mA cm^–2^, 0.5 mAh cm^–2^) was investigated by using a high‐resolution transmission electron microscope (HRTEM). As shown in Figure 3e, many nanodots with a size of 5–10 nm can be found on the porous carbon framework, which is attributed to the RP anchored on the carbon host after the vaporization–condensation–conversion process. Meanwhile, the surface showed a homogeneous crystal orientation with the lattice spacing of 0.250 nm (Figure 3f). Remarkably, the lattice spacing increased from 0.247 to 0.25 nm, which is related to the formation of ZnP solid solution alloy.^[^ ^13^, ^24^ ^]^ Therefore, the morphological evolution of Zn plating on the Zn@RP‐NC electrode could be refined into a schematic diagram as shown in Figure 3g. The surface morphology became denser with the increase of Zn deposition. At a fixed capacity of 0.5 mAh cm^–2^, a homogeneous ZnP alloy interface layer was basically formed. In the subsequent deposition process, benefiting from the regulation of Zn‐ion flux by the interface layer, the excellent conductivity of the NC achieved the dendrite‐free Zn deposition. Besides, the surface composition on Zn@ZnP‐NC anode after plating 0.5 mAh cm^–2^ was investigated using XPS facilitated by Ar^+^ sputtering (Figure 3h and Figure S14 (Supporting Information)). Both the top and depth Zn 2p spectra represent two strong peaks of Zn 2p_3/2_ and Zn 2p_1/2_ that correspond to the energy peaks of 1021.8 and 1045.2 eV,^[^ ^11a,b^ ^]^ indicating the uniform distribution of Zn atoms in the vertical direction. For the P 2p spectra, the fitted peaks at 139.9 and 136.2 eV are related to the Zn 3s and P 2p. The other small peak at 134.2 eV only existed in the top surface, which is attributed to the oxidation of the surface.^[^ ^12^, ^25^ ^]^ In the C 1s spectrum at 0 min sputtering, three peaks at 284.6, 285.6, and 288.5 eV indicated the presence of C═C/C—C, C—N, and C═O, respectively.^[^ ^14b,c^ ^]^ Notably, as the Ar^+^ sputtering increased to 5 min, the C—O—P and C═O bonds appeared at 290.4 and 287.0 eV, respectively. Upon further sputtering to 10 min, the P—C bond appeared at 283.4 eV,^[^ ^14a,d^ ^]^ which displayed a similar composition to the Zn@RP‐NC surface. Therefore, these results indicate that the ZnP alloy protective layer was formed on the surface of the electrode, which can adjust the Zn‐ion flux and achieve the uniform Zn deposition.

**Figure 4:**
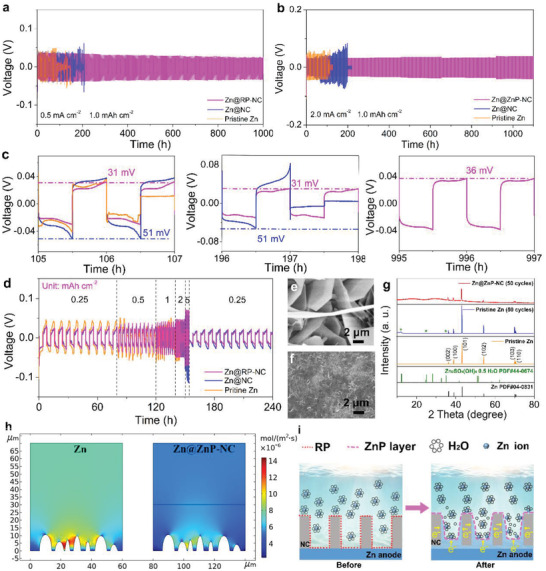
Voltage profiles of the pristine Zn, Zn@RP‐NC, and Zn@ZnP‐NC symmetric batteries at the current densities of a) 0.5 mA cm^–2^ and b) 2.0 mA cm^–2^ with a capacity of 1.0 mAh cm^–2^, and c) magnified voltage‐time curves at different cycles in (b). d) Rate performance of Zn symmetric batteries. e,f) SEM images and g) XRD patterns of the pristine Zn and Zn@ZnP‐NC electrodes after 50 cycles at a current density of 0.5 mA cm^–2^. h) Simulation results of the Zn‐ion flux distribution on the surface of the Zn@ZnP‐NC electrode. i) Schematic depiction of Zn deposition behavior on Zn@ZnP‐NC anode.

The electrochemical performance of the electrodes for Zn plating/stripping in symmetric batteries with a capacity of 1.0 mAh cm^–2^ is shown in **Figure** **4** and Figure S15 of Supporting Information. At a low current density of 0.5 mA cm^–2^ (Figure 4a), the pristine Zn and Zn@NC symmetric batteries showed abrupt failure after 90 and 150 h, respectively. In contrast, benefiting from the ZnP‐NC protective layer, the Zn@RP‐NC symmetric battery displayed highly reversible and stable plating/stripping for 1000 h. The pristine Zn symmetric battery revealed irregular overpotential and fast short circuits at ≈100 h with the current densities of 1.0 (Figure S15a of Supporting Information) and 2.0 mA cm^–2^ (Figure 4b), respectively. The Zn@NC symmetric batteries also exhibited a short circuit fault at ≈200 h under the same test conditions. Obviously, the NC is a host for Zn deposition and the NC cannot effectively inhibit the dendrite growth, which can only delay the battery short circuit in a short time. This result can be predicted in the SEM image analysis, as can be seen in Figure S10 of Supporting Information. In remarkable contrast, the Zn@RP‐NC symmetric batteries maintained a long and stable plating/stripping for more than 500 h at the current density of 1.0 mA cm^–2^. Meanwhile, the Zn@RP‐NC symmetric battery displayed the long‐term stable plating/stripping for 1100 h at the current density of 2.0 mA cm^–2^. Figure S16a,b (Supporting Information) displays the NOP of the pristine Zn, Zn@NC, and Zn@RP‐NC at the current densities of 0.5 and 2.0 mA cm^–2^, respectively. The Zn platings on pristine Zn and Zn@NC electrodes were measured to be 48 and 34 mV at the current density of 0.5 mA cm^–2^, respectively. When the current density increased to 2.0 mA cm^–2^, the corresponding NOP values of the two electrodes were 69 and 57 mV, respectively. Notably, the values of the Zn@RP‐NC electrode were consistent between those of the Zn and Zn@NC electrodes, which were 40 and 63 mV, respectively. The relatively large deposition voltage reflected that the deposited Zn metal was small and dense.^[^ ^18c^ ^]^ Meanwhile, the voltage profile detail of symmetric batteries under the current density of 2.0 mA cm^–2^ showed that the overpotential of the Zn@RP‐NC electrode remained ≈30 mV, lower than those of the pristine Zn and Zn@NC electrodes (Figure 4c). This is attributed to the stable interface on the Zn anode.^[^ ^2a^ ^]^ With a high current density of 5.0 mA cm^–2^ (Figure S15b of Supporting Information), the Zn@RP‐NC electrode could also deliver a reversible and stable plating/stripping for more than 300 h than the Zn@NC (130 h) and pristine Zn (65 h) electrodes. The Zn@RP‐NC electrode also displayed a small overpotential of 53 mV after 150 cycles (Figure S15c of Supporting Information) and 61 mV after 340 cycles (Figure S15d of Supporting Information). Even at the current densities of 1.0 mA cm^–2^ with a capacity of 3.0 mAh cm^–2^ (Figure S17a of Supporting Information) and 2.0 mA cm^–2^ with a capacity of 2.0 mAh cm^–2^ (Figure S17b of Supporting Information), the Zn@RP‐NC electrode could also operate over 270 h, implying the durability of the protective layer. The rate performances of the symmetric batteries are compared in Figure 4d. The Zn@RP‐NC electrode displayed lower overpotentials and flatters during the plating/stripping processes than the pristine Zn and Zn@NC during all the rate profiles. Furthermore, the Zn@RP‐NC anode exhibited a better performance in long‐term cycling and high current density than some recently reported Zn anodes (Figure S18 and Table S1 of Supporting Information).^[^ ^1^, ^4^, ^26^ ^]^ The excellent electrochemical performance can be attributed to the formed ZnP protective layer on the electrode surface, which effectively regulates the Zn‐ion flux. Meanwhile, the 3D conductive NC realizes the uniform distribution of the electric field on the Zn anode surface.

During the long‐term plating/stripping process, the structural stability of the artificial protective layer plays a crucial role in dendrite‐free Zn deposition. Therefore, the pristine Zn and Zn@ZnP‐NC electrodes were tested after 50 cycles at a current density of 0.5 mA cm^–2^. Figure 4e and Figure S19a of the Supporting Information show the Zn deposition on the pristine Zn electrode. The uneven and random distribution of flaky Zn could cause uneven electric field distribution on the electrode surface and Zn‐ion concentration at the interface, further intensifying the growth of dendrites.^[^ ^4c^ ^]^ The SEM images of the Zn@ZnP‐NC displayed a smooth deposition morphology (Figure 4f and Figure S19b of the Supporting Information), which indicated that the presence of the ZnP alloy protective layer could achieve uniform Zn deposition. As shown in Figure S20 of Supporting Information, the maximum height difference of the pristine Zn electrode surface was ≈70 µm (Figure S20a,b of the Supporting Information), which was more than that of the Zn@ZnP‐NC electrode (≈30 µm, Figure S20c,d of the Supporting Information). The cross‐sectional SEM image and their corresponding EDS mapping images of Zn@ZnP‐NC after 50 cycles at a current density of 0.5 mA cm^–2^ are shown in Figure S21 of the Supporting Information. There was no obvious structural change in the Zn@ZnP‐NC electrode, which indicated that the ZnP‐NC interfacial layer had excellent stability. These phenomena indicate that the ZnP‐NC interfacial layer has excellent stability. Meanwhile, the hydrogen evolution can induce the formation of loose and brittle Zn_4_SO_4_(OH)_6_·0.5H_2_O on the surface of the Zn electrode, resulting in low CE and severe capacity fading.^[^ ^4^, ^27^ ^]^ Therefore, to verify that the protective layer could effectively suppress the hydrogen evolution reaction, the surface by‐products of the pristine Zn and Zn@ZnP‐NC electrodes were further monitored by XRD (Figure 4g). Compared to the Zn anode, no obvious peaks were detected in the Zn@ZnP‐NC electrode, which indicated that the formed dense and smooth ZnP‐NC layer could suppress the hydrogen evolution reactions on the electrode interfaces. Furthermore, to further explore the uniform Zn deposition behavior with the artificial interface layer toward a stable Zn anode, the distribution of Zn‐ion flux on the pristine Zn electrode and the Zn@ZnP‐NC electrode was further monitored by COMSOL Multiphysics software (Figure 4h). The protrudes on the planner substrate were used to simulate the roughness of the surface of the Zn foil.^[^ ^26i^ ^]^ For the pristine Zn electrode, Zn ions tended to be accumulated near the protrusion during the plating progress. These Zn protrusions could further induce a “tip effect”, causing uneven depositions and consequent dendrite growth on the electrode surface.^[^ ^1^, ^27^ ^]^ Corresponding to the pristine Zn model, red areas could be seen near the protrusions. These areas, called “hot spots,” contained large Zn‐ion flux that will provide conditions for dendrite growth.^[^ ^28^ ^]^ Conversely, when the ZnP‐NC coating layer was introduced into the Zn surface, the Zn‐ion flux was effectively redistributed and homogenized. Meanwhile, no “hot spots” were found on the surface of the Zn electrode, which indicated that a uniform Zn‐ion flux was achieved under the regulation of the ZnP‐NC interfacial layer. Therefore, Figure 4i further reveals the deposition behavior of the Zn ions on the Zn@ZnP‐NC. Before plating, the Zn@RP‐NC electrode exhibited weak hydrophilicity, which resulted in an obvious ion concentration gradient on the electrode surface. Subsequently, the RP nanoparticles anchored on the 3D carbon framework formed a ZnP alloy layer during the initial Zn deposition process. The formed alloy layer enhanced the hydrophilicity of the electrode surface, and the ion concentration gradient was gradually uniform on the electrode surface. Furthermore, the diffusion mechanism of the Zn ions on the electrode surface gradually changed from 2D to 3D diffusion. Thanks to the excellent electrical conductivity of the 3D carbon skeleton and high ionic conductivity of the ZnP‐NC layer, the even electric field on the electrode surface made the Zn ions that passed through the alloy layer, which could easily obtain electrons and formed Zn atoms, thereby eliminating the “tip effect” and realizing the uniform distribution of Zn ions.

Figure S22 of Supporting Information shows the CE of Zn plating/stripping in Zn//Ti@RP‐NC, Zn//Ti@NC, and Zn//Ti batteries at the current densities of 0.5 and 1.0 mA cm^–2^. The Zn//Ti@RP‐NC battery could deliver an average Coulombic efficiency (ACE) of 97.3% over 80 cycles at 0.5 mA cm^–2^. However, the fluctuated CE of Zn//Ti and Zn//Ti@NC batteries appeared at 30 and 50 cycles, respectively due to the accumulation of dead Zn and the formation of dendrites as shown in Figure S22a of Supporting Information.^[^ ^4^, ^19^ ^]^ Meanwhile, the AEC of Zn//Ti@RP‐NC battery was 98.2% after 100 cycles at 1.0 mA cm^–2^ (Figure S22b of Supporting Information). Additionally, optical microscopy was employed to in situ monitor the Zn metal plating process on the pristine Zn and Zn@RP‐NC electrodes. Some Zn protrusions were first observed at the groove position on the Zn surface within 10 min, and they were continuously accumulated into the mossy‐like Zn dendrites after 40 min (Figure S23a of Supporting Information). The heterogeneous electric field distribution at the electrode/electrolyte interface is caused by surface imperfections, which increases the local current density, resulting in uneven nucleation and Zn dendrite growth.^[^ ^1^, ^3^, ^4^ ^]^ Comparatively, the dendrite‐free Zn deposition induced by uniformly distributed Zn ions can be found on the Zn@RP‐NC electrode during the Zn plating for 40 min as shown in Figure S23b of Supporting Information.

**Figure 5:**
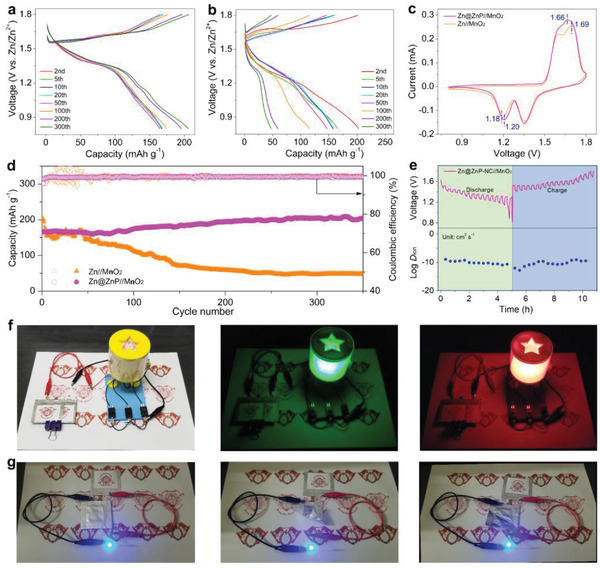
Charge/discharge curves of the a) Zn@ZnP‐NC//MnO_2_ and b) Zn//MnO_2_ full cells. c) CV curves and d) cycle performance of the Zn@ZnP‐NC//MnO_2_ and Zn//MnO_2_ full cells. e) GITT curve and the corresponding ion diffusivity of the Zn@ZnP‐NC//MnO_2_ full cell. f,g) Optical images of the Zn@ZnP‐NC//MnO_2_ pouch cell and the working states.

In Mn‐based oxides as cathode materials, the unique tunnel structure facilitates the transport of Zn ions, and they have been extensively studied for AZIBs.^[^ ^29^ ^]^ To test the practical application of the Zn@ZnP‐NC anode, the full cell using MnO_2_ as a cathode was assembled. Figure S24 of Supporting Information shows the SEM images of MnO_2_, which was self‐assembled into a 3D structure from slender nanowires. As shown in **Figure** **5**a, the discharge capacity of the Zn@ZnP‐NC//MnO_2_ cell in the second cycle was 173 mAh g^–1^ at the current density of 1.0 A g^–1^ and remained stable in subsequent 350 cycles. In contrast, the discharge capacity of the Zn//MnO_2_ cell in the second cycle was 204 mAh g^–1^, whereas the capacity decayed rapidly in subsequent cycles (Figure 5b). The CV curves of the Zn@ZnP‐NC//MnO_2_ and Zn//MnO_2_ cells are shown in Figure 5c. The Zn@ZnP‐NC//MnO_2_ cell displayed higher reduction potential and lower oxidation potential than the Zn//MnO_2_ cell, which indicated that the ZnP alloy protective layer improved the reversibility of the electrode.^[^ ^2b^ ^]^ As shown in Figure 5d, the Zn@ZnP‐NC//MnO_2_ cell maintained a reversible capacity of 203 mAh g^–1^ after 350 cycles. The possible reason for the slight capacity increase is that the preadded MnSO_4_ is deposited on the cathode and provides ex‐active sites.^[^ ^18^, ^30^ ^]^ However, the capacity of the Zn//MnO_2_ cell was continuously decreased after 50 cycles and delivered an extremely low capacity of 48 mAh g^–1^ at the end of cycling. This means that the pristine Zn anode suffers from severe corrosion and side reactions. The excellent electrochemical performance of the Zn@ZnP‐NC//MnO_2_ cell implies that the ZnP alloy protective layer not only delays electrode corrosion but also effectively prevents side reactions.

The interfacial impedance of the Zn@ZnP‐NC//MnO_2_ and Zn//MnO_2_ cells were studied using EIS tests after the 10th and 100th cycles, as shown in Figure S25a,b of Supporting Information, respectively, to understand the difference in the cell performance. Although the resistances of the two cells gradually increased with cycling, the semicircles in the high‐medium frequency region of the Zn@ZnP‐NC//MnO_2_ cell were smaller than that of the Zn//MnO_2_ cell, which indicated that the Zn@ZnP‐NC//MnO_2_ had lower SEI film resistance and charge transfer resistance at the electrode and electrolyte interface.^[^ ^1^, ^31^ ^]^ Meanwhile, the relationship of *Z*′ versus *ω* ^–1/2^ in the low‐frequency region is shown in Figure S25c,d of Supporting Information. At either the 10th or the 100th cycle, the Zn@ZnP‐NC//MnO_2_ cell represented lower slopes than the Zn//MnO_2_ cell, which indicated its excellent Zn‐ion kinetics. The reaction kinetics of the full cells were further evaluated by the galvanostatic intermittent titration technique (GITT). As shown in Figure 5e, the calculated *D*_ion_ values of Zn@ZnP‐NC//MnO_2_ cell were higher than that of Zn//MnO_2_ cell (Figure S26 of Supporting Information), which indicated fast Zn‐ion diffusion kinetics in the Zn@ZnP‐NC//MnO_2_ cell. The feedback result is consistent with the EIS. A pouch cell was further assembled to prove the practical application as shown in Figure S27a of Supporting Information. The Zn@ZnP‐NC//MnO_2_ cell delivered an open circuit potential of 1.43 V (Figure S27b Supporting Information). As shown in Figure 5f, red and green electronic devices could be easily lightened by two Zn@ZnP‐NC//MnO_2_ cells connected in series. In addition, the brightness of the blue light‐emitting diode (3.0 V) remained unchanged even in the structural distortion and recovery experiments (Figure 5g), which indicated that the Zn@ZnP‐NC//MnO_2_ cell had the potential for wearable applications. These phenomena highlight their great potential in wearable electronic products and provide us with valuable experience in the later development of flexible electronic devices.

## 3 Conclusions

In summary, we fabricated the ZnP‐NC artificial protective layer on the Zn metal surface and successfully achieved the dendrite‐free Zn deposition. The synergistic effect of the ZnP alloy artificial protective layer and the carbon framework enabled a uniform electric field strength distribution and a homogeneous Zn‐ion flux on the electrode surface, resulting in a long‐term stable Zn plating/stripping. In addition, the quantitative electroplating of Zn on the electrode surface revealed the dynamic evolution mechanism of the ZnP alloy artificial protective layer. As a result, the Zn@ZnP‐NC anode realized a long‐term (1100 h) dendrite‐free Zn plating/stripping at 2.0 mA cm^–2^/1.0 mAh cm^–2^. Furthermore, the Zn@ZnP‐NC//MnO_2_ full cell delivered a stable capacity of about 200 mAh g^–1^ after 350 cycles at 1.0 A g^–1^. This strategy is expected to open a way in the development of dendrite‐free Zn metal anode, and provides a reference for the design of high‐performance rechargeable AZIBs.

## Conflict of Interest

The authors declare no conflict of interest.

## Supporting information

Supporting information

## Data Availability

The data that support the findings of this study are available from the corresponding author upon reasonable request.
