# Supplementary material for: Regulating Dendrite‐Free Zinc Deposition by Red Phosphorous‐Derived Artificial Protective Layer for Zinc Metal Batteries
Source: Adv Sci (Weinh). 2022 Apr 24;9(18):2200155. doi: 10.1002/advs.202200155 (PMC9218763; doi:10.1002/advs.202200155)
Supplement: Supplementary file 1 — Supporting information [file ADVS-9-2200155-s001.pdf]

Supporting Information

Regulating Dendrite-Free Zinc Deposition by Red Phosphorous-Derived Artificial Protective Layer for Zinc Metal Batteries

*Tian Wang, Qiao Xi, Yifan Li, Hao Fu, Yongbin Hua, Edugulla Girija Shankar, Ashok Kumar Kakarla, and Jae Su Yu**

T. Wang, Y. Hua, E. G. Shankar, A. K. Kakarla, J. S. Yu
Department of Electronics and Information Convergence Engineering, Institute for Wearable Convergence Electronics, Kyung Hee University, Yongin-si, Gyeonggi-do 17104, Republic of Korea
E-mail: jsyu@khu.ac.kr (J. S. Yu)

Q. Xi, Y. Li
Frontiers Science Center for Flexible Electronics (FSCFE), Shaanxi Institute of Flexible Electronics (SIFE), Northwestern Polytechnical University, 127 West Youyi Road, Xi'an 710072, China

H. Fu
Department of Physics, Dongguk University, Seoul 04620, Republic of Korea

Experimental Section

Preparation of NC: 0.02 g of acid-treated multiwall carbon nanotubes (CNTs) was dispersed in 150 mL de-ionized (DI) water. Then, 1.0 g thiourea was added to the CNTs dispersion and stirred for 1 h. Afterwards, the water was removed by rotary evaporation and the collected mixture was thermal treated at 750 °C with a rate of 2 °C min^{-1} for 4 h under an atmosphere of argon (Ar). Finally, the NC was obtained by washing with DI water and ethanol, and drying at 60 °C in vacuum oven for overnight.

Preparation of RP-NC: RP-NC coating layer was prepared by the vaporization-condensation-conversion process. The mixture NC (100 mg) and RP powder (40 mg) were sealed in a quartz tube and the sealed quartz tube put into a tube furnace, and then it was heated to 500 ^{o}C with a rate of 2 °C min^{-1} for 4 h under Ar atmosphere. Subsequently, the temperature was cooled down to 260 ^{o}C and kept for 2 h to convert white P to RP, while the cooling rate was 1 ^{o}C min^{-1}. Finally, the as-prepared product was obtained after cooling down to room temperature.

Preparation of Zn@RP-NC, Zn@NC, Ti@RP-NC, and Ti@NC: At first, Zn foil with a thickness of 0.02 mm was sonicated in ethanol to clean the surface. For Zn@RP-NC anode, a slurry of RP/NC and polyvinylidene fluoride (PVDF) in N-methylpyrrolidone (NMP) was employed onto the Zn foil by a slurry coating method and dried at 60 °C in a vacuum oven for 12 h. As a comparison sample, the Zn@NC was prepared by the same method. A similar method was used to prepare Ti@RP-NC and Ti@NC with Ti foil (0.02 mm thickness).

Preparation of MnO_2: In a typical synthesis, $MnSO_4\cdot H_2O$ (0.67 g) and $(NH_4)_2S_2O_8$ (0.91 g) were added to DI water and stirred for 1 h. After that, the solution was heated at 140 ^{o}C for 14 h. Finally, the as-prepared product was washed with DI water and ethanol individually. The MnO_2 electrode was fabricated via the slurry coating method. The MnO_2, super-P, and PVDF with a mass ratio of 75:15:10 were added in NMP to form a homogeneous slurry, which was then coated onto the Ti foil and dried at 60°C for 12 h in a vacuum oven. The Ti foil was punched into circle disks with a diameter of 12 mm. The MnO_2 loading for each disk was controlled around 0.45-0.55 mg.

Material Characterization: Micro-morphologies of materials and electrodes were observed by using a field-emission scanning electron microscope (FE-SEM; MERLIN (Carl Zeiss)) and a transmission electron microscope (TEM; FEI Talos F200i). The energy-dispersive X-ray spectroscopy (EDS) was utilized to detect the elements. The 3D morphology and surface roughness were characterized by confocal laser microscopy (KEYENCE VK-X200). The crystal structures were analyzed by X-ray diffraction (XRD, D8 Advance (Bruker)) with monochromatic Cu Ka in the 2θ range of 5-70°. The component and valence states of elements

were evaluated by the X-ray photoelectron spectroscopy (XPS, K-alpha (Thermo Electron)). Binding energy corrections were made to the raw spectra by using the C 1s peak at 284.6 eV. The Zn dendrite growth was in-situ observed by using an optical microscope.

Fabrication of Batteries and Electrochemical Measurements: The symmetrical cells were assembled to investigate the electrochemical behavior of the pristine Zn, Zn@RP-NC, and Zn@RP-NC electrodes. The electrode was cut into discs with a diameter of 12 mm. The electrolyte was 2 M $ZnSO_4$ solution. Glass fiber as a separator was employed to assemble standard two-electrode Swagelok cell. The same electrolyte was used in each battery to get standard test results. Cyclic voltammetry (CV) curves of Zn plating/stripping were measured at a scan rate of 5 mV s^{-1} in a three-electrode system consisting of Ti plate, Zn plate, and Ag/AgCl (saturated KCl) as working, counter, and reference electrodes, respectively. Linear polarization was measured at 1 mV s^{-1} in three-electrode configuration. The Zn//MnO_2 full cell was prepared by the Zn@RP-NC (Zn deposition on the Zn@RP-NC electrode with a capacity of 0.5 mAh cm^{-2}) anode and MnO_2 cathode, which was separated by a glass fiber in CR2032 coin cells. 2 M $ZnSO_4$ and 0.1 M $MnSO_4$ aqueous solutions were used as the electrolyte, in which the $MnSO_4$ can inhibit the dissolution of Mn^{2+} from MnO_2.[1] These full cells were cycled between 0.8 and 1.8 V. The measurements of charge/discharge and GITT were carried out on a WonaTech Automatic cell test instrument (WBCS3000). The CV curves with a voltage of 0.8-1.8 at 0.1 mV s^{-1} and electrochemical impedance spectroscopy (EIS) plots in a frequency range from 100 kHz to 1.0 mHz were measured on the Iviumstat electrochemical workstation.

Computational Simulation: A simplified 2D deposition model at the interface between the Zn metal anode and electrolyte was built, which coupled the Nernst-Planck equation and was solved by COMSOL Multiphysics using the finite element method to display the inhibitory effect of the protective layer in Zn dendrite growth. The length of the electrode was 60 µm. The protrudes on the planner substrate were used to simulate the roughness of the surface of the Zn foil. The voltage excitation between the electrode and the electrolyte was 200 mV. The Zn@ZnP-NC electrode had a protective layer with a thickness of ~15 µm and the ionic conductivity was 1.207×10^{-4} S cm^{-1}. Meanwhile, the ionic conductivity of the 2 M $ZnSO_4$ electrolyte was ≈5 S m^{-1}.[2]

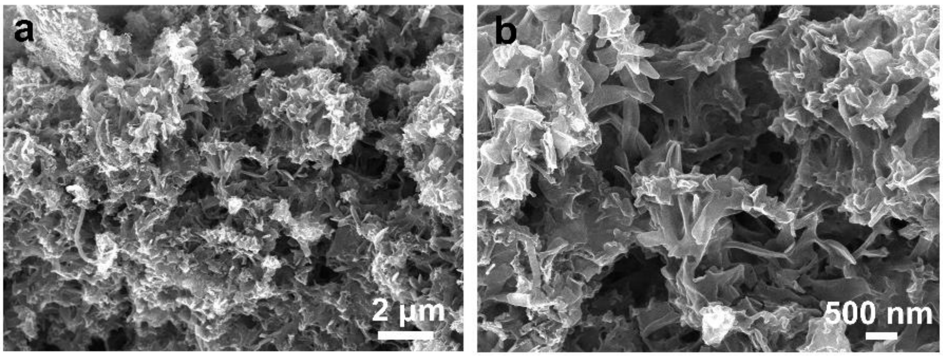

Figure S1. (a) Low- and (b) high-magnification SEM images of NC.

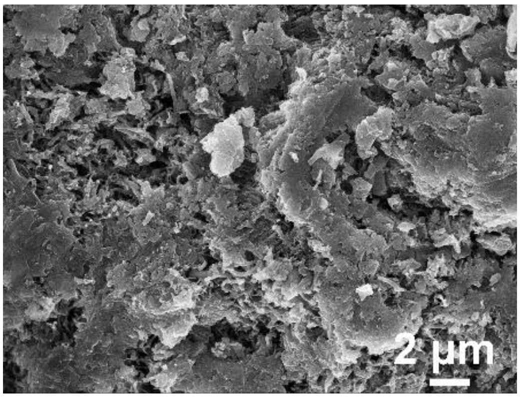

Figure S2. SEM image of RP-NC architecture.

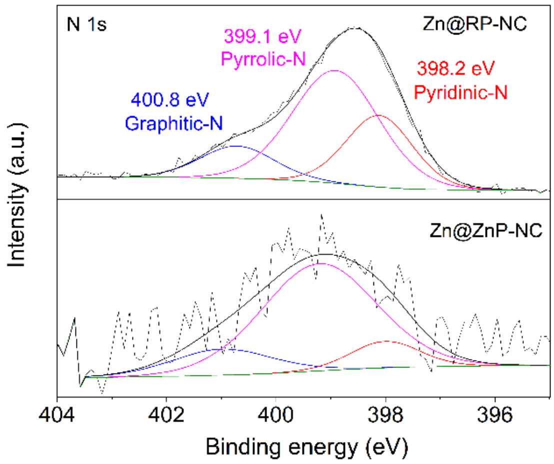

Figure S3. High-resolution XPS spectra of N 1s.

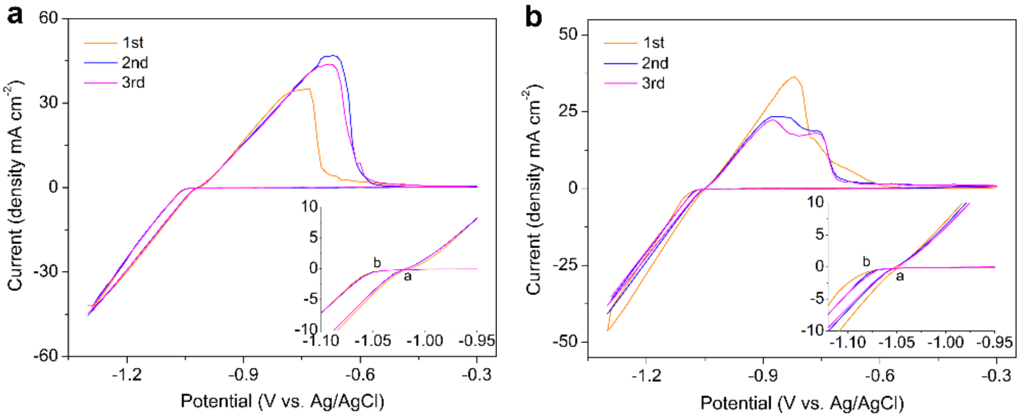

Figure S4. CV curves of Zn plating/stripping on the (a) Ti@NC and (b) Ti foil.

The CV curves were measured with Ti, Zn foil, and Ag/AgCl as the working, counter and reference electrode, respectively. The point a is the crossover potential. The point b is the potential of Zn ion, which means the Zn ions start to be reduced. The potential value between the a and the b represents the NOP, which can be used to evaluate the extent of electrode polarization.[1b]

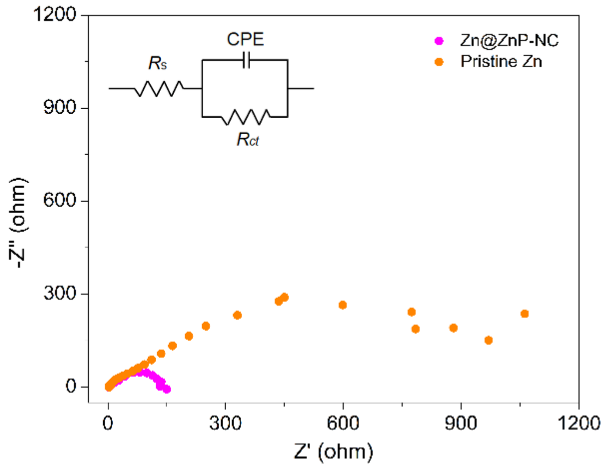

Figure S5. EIS plots of the Zn@ZnP-NC and pristine Zn symmetric batteries.

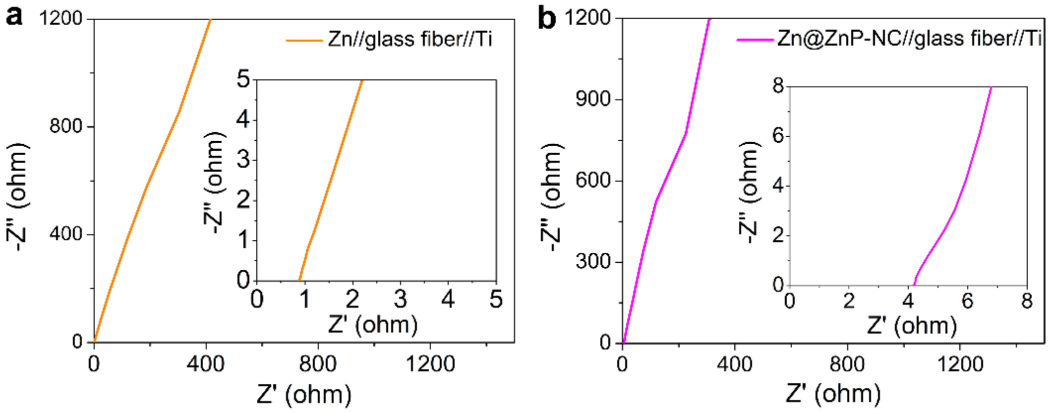

Figure S6. Nyquist plots tested at open circuit voltage of (a) Zn//Ti and (b) Zn@ZnP-NC//Ti configurations with glass fiber as separator. (inset: enlargement of the indicated curve). The ion conductivity (σ) was calculated by the following equation:

$$\sigma = \frac{L}{R_b S}$$

where L is the thickness of the ZnP-NC layer, R_b represents the resistance of the coating layer, and S is the effective contacting area.[3]

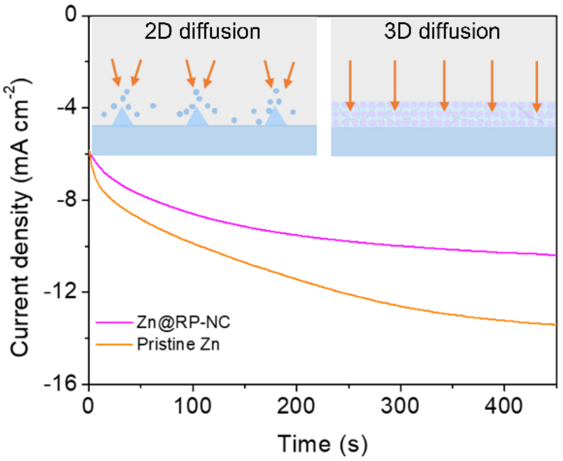

Figure S7. Chronoamperometry curves of pristine Zn and Zn@RP-NC at -150 mV overpotential.

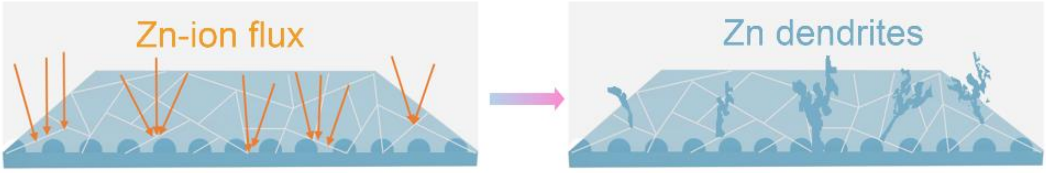

Figure S8. Schematic diagram of Zn deposition on the pristine Zn electrode.

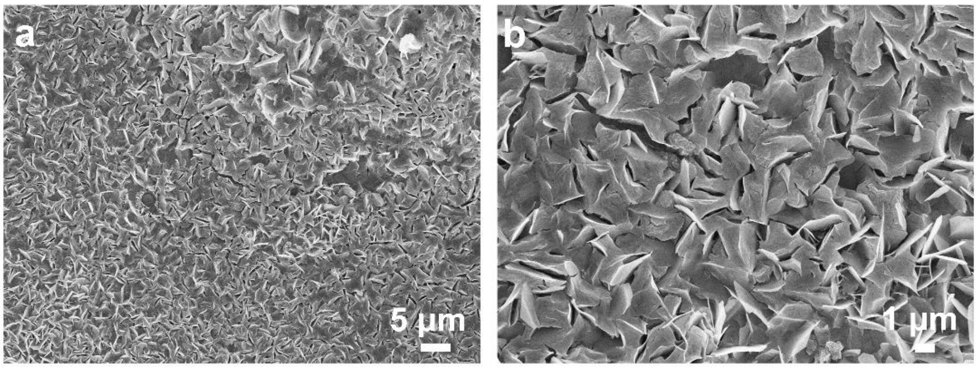

Figure S9. SEM images of the pristine Zn electrode after plating of 0.5 mAh cm^{-2}.

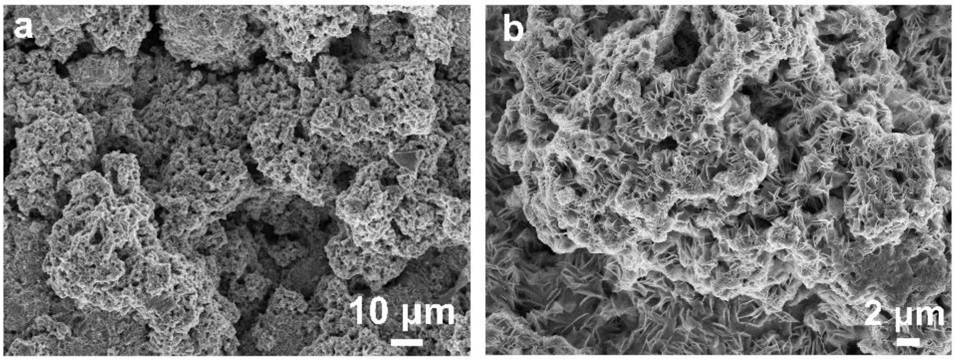

Figure S10. SEM images of the Zn@NC electrode after plating of 0.5 mAh cm^{-2}.

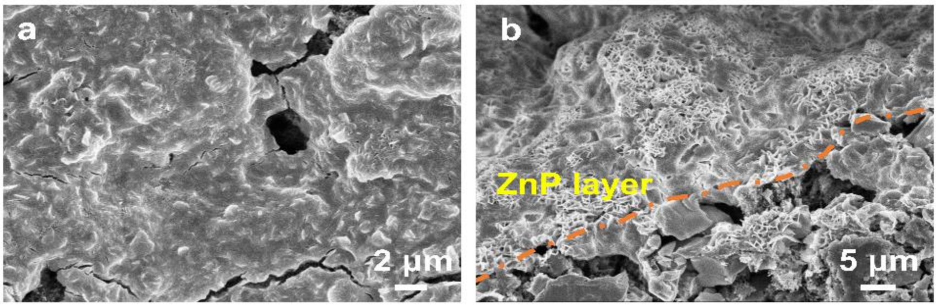

Figure S11. (a) Top-view and (b) cross-sectional SEM images of the modified electrode after plating of 0.5 mAh cm^{-2}.

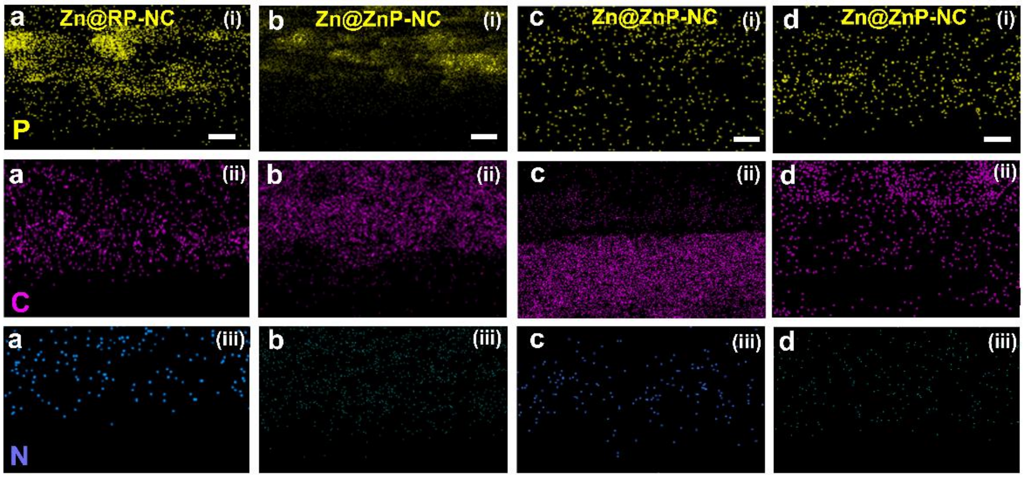

Figure S12. Corresponding EDS mapping images of (a)(i,ii,iii) the fresh Zn@RP-NC electrode and the Zn@RP-NC electrodes, (b)(i,ii,iii) after plating of 0.5 mAh cm^{-2}, (c)(i,ii,iii) after plating of 1.0 mAh cm^{-2}, and (d)(i,ii,iii) after plating of 3.0 mAh cm^{-2}.

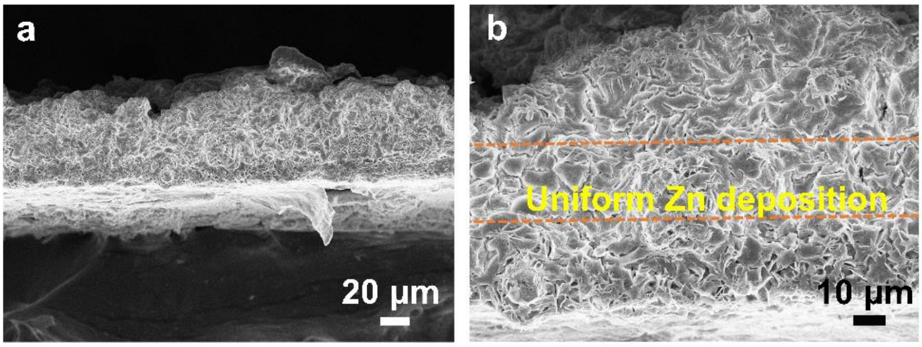

Figure S13. (a,b) Cross-sectional SEM images of the modified electrode after plating of 3.0 mAh cm^{-2}.

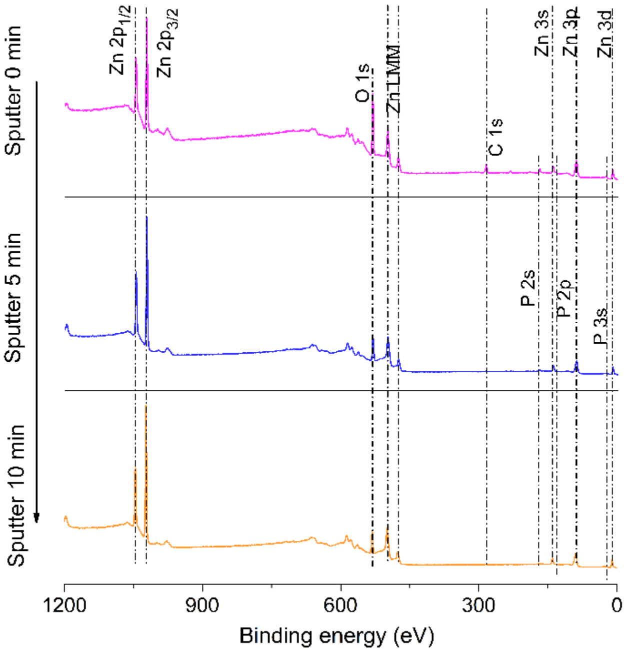

Figure S14. XPS spectra of the Zn@ZnP-NC electrode after sputtering for 0, 5, and 10 min.

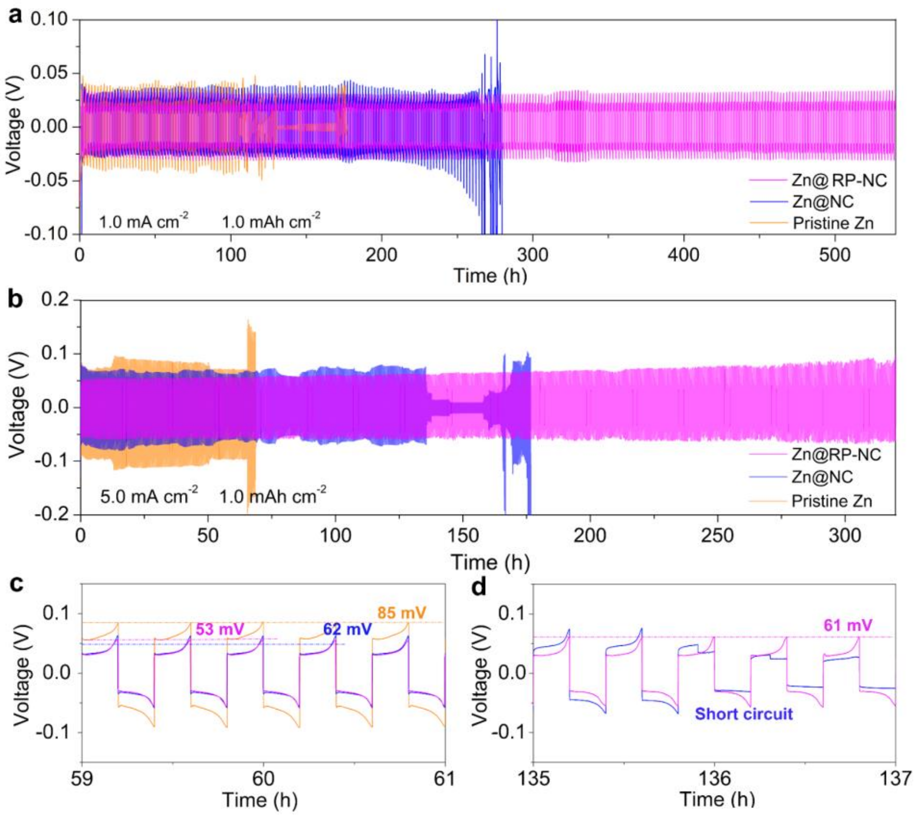

Figure S15. Comparative cycling stability of the Zn@RP-NC, Zn@NC, and pristine Zn symmetric batteries at (a) 1.0 mA cm^{-2} and (b) 5.0 mA cm^{-2} with a plating/stripping capacity of 1.0 mAh cm^{-2}. (c,d) Magnified voltage-time curves at different cycles in (b).

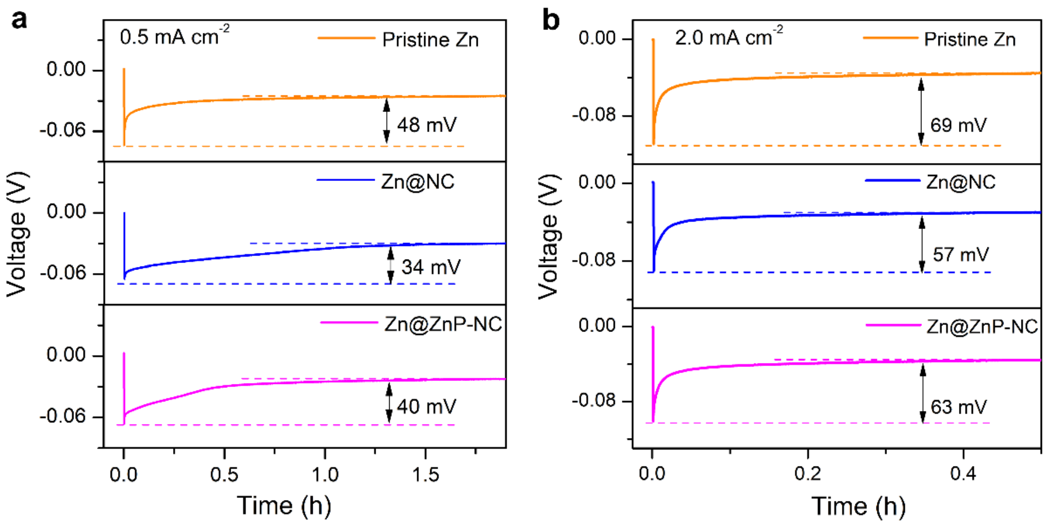

Figure S16. Voltage-time curves of Zn plating on pristine Zn, Zn@NC, and Zn@RP-NC at the current densities of (a) 0.5 and (b) 2.0 mA cm$^{-2.}$

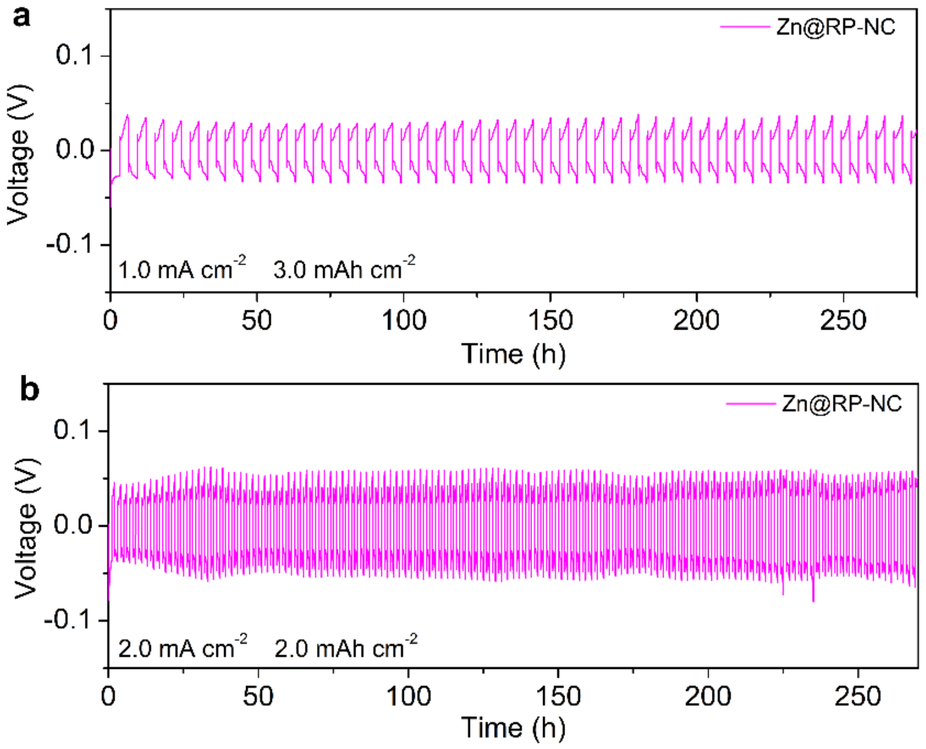

Figure S17. Cycling performance of Zn@RP-NC symmetric batteries under the current densities and capacities of (a) 1.0 mA cm^{-2} with 3.0 mA h cm^{-2} and (b) 2.0 mA cm^{-2} with 2.0 mA h cm^{-2}.

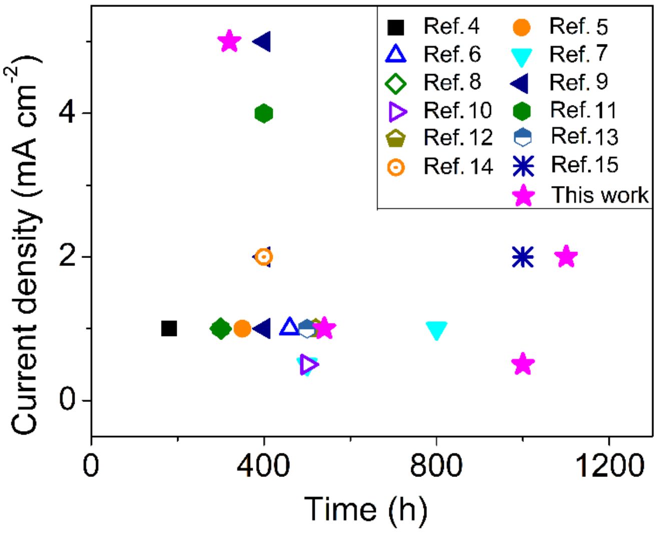

Figure S18. Comparative electrochemical performance with other reported literatures with different coating layers.

Table S1. Cycling performance of some Zn symmetric batteries by modification of interfacial layer with different strategies.

**Interfacial layer**	Method	Current desiity (mA cm^{-2})	Capacity (mAh cm^{-2})	Time (h)	Ref.
**PAM modified interface**	Galvanostatic electrodeposition	1.0	1.0	180	[4]
**Ag-Zn**	Replacement reaction	1.0	1.0	350	[5]
**Faceted TiO_2 coated**	Slurry coating	1.0	1.0	460	[6]
**3D ZnF_2**	Electrodeposition	0.5	1.0	500	[7]
		1.0	1.0	800	
**MXene@Zn**	Electrodeposition	1.0	1.0	300	[8]
**Zn@Mg-Al LDH**	Slurry coating	1.0	1.0	400	[9]
		2.0	1.0	400	
		5.0	1.0	400	
**Zn/Sn(200)**	Chemical displacement reaction	0.5	1.0	500	[10]
**In-coated Zn**	Ion-exchange	1.0	1.0	300	[11]
		4.0	1.0	400	
**Zn|In**	Spontaneous galvanic replacement	1.0	1.0	520	[12]
**Zn(002)**	Large rolling deformation	1.0	1.0	500	[13]
**502 glue coated Zn**	Spin-coating	2.0	1.0	400	[14]
**Stratified deposition framework**	**redox reaction +sputter**	2.0	1.0	1000	[15]
**Zn@ZnP-NC**	Slurry coating	0.5	1.0	1000	This work
		1.0	1.0	540	
		2.0	1.0	540	
		5.0	1.0	320	

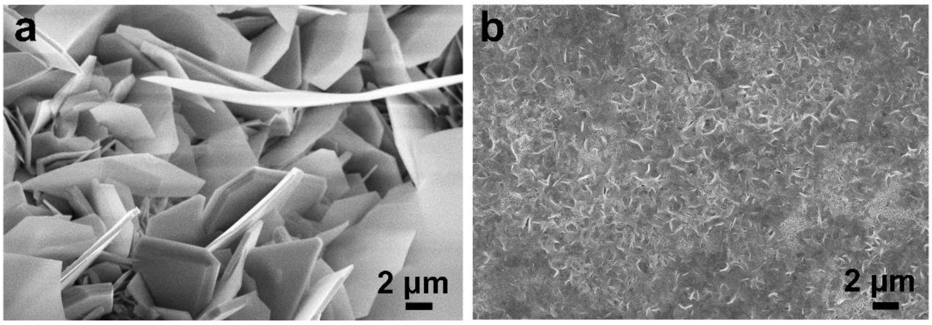

Figure S19. SEM images of the pristine Zn and Zn@ZnP-NC samples after 100 h at a current density of 0.5 mA cm^{-2}.

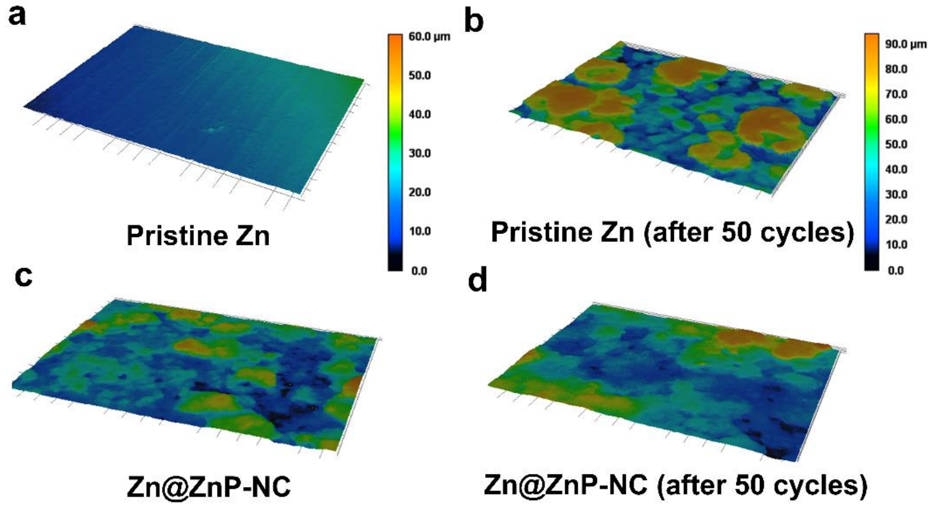

Figure S20. 3D confocal microscope images of (a) the pristine Zn, (b) Zn electrode after 50 cycles, (c) Zn@ZnP-NC, and (d) Zn@ZnP-NC electrode after 50 cycles.

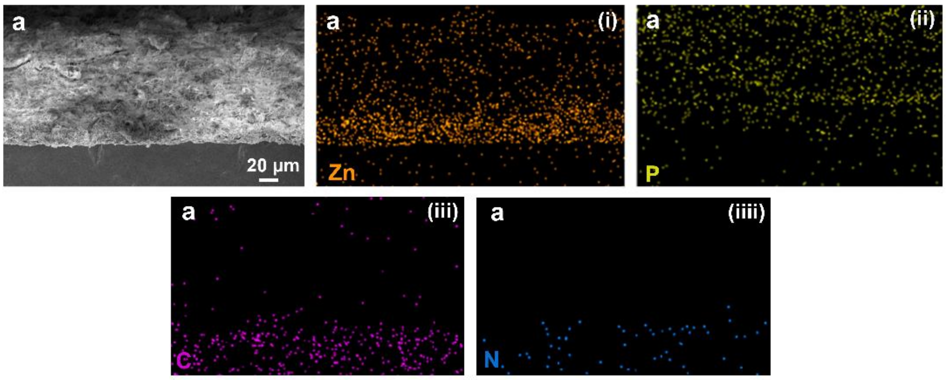

Figure S21. Cross-sectional SEM image and the corresponding EDS mapping images of Zn@ZnP-NC after working 100 h at a current density of 0.5 mA cm^{-2}.

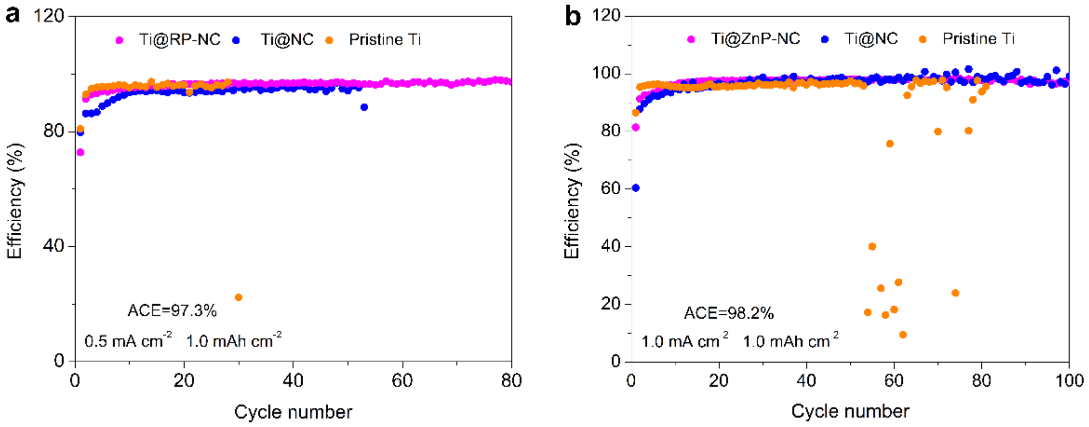

Figure S22. Coulombic efficiency of the Zn//Ti@RP-NC, Zn//Ti@NC, and Zn//Ti batteries at (a) 0.5 mA cm^{-2} and (b) 1.0 mA cm^{-2}.

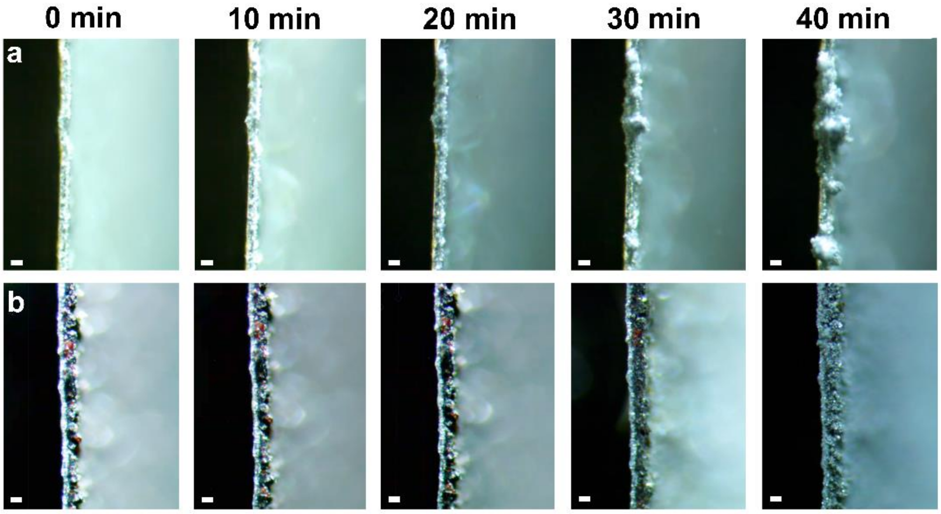

Figure S23. In-situ optical visualization observations of the (a) pristine Zn anode and (b) Zn@RP-NC anode before and after gradually plating Zn metal for 10, 20, 30, and 40 min at a current density of 2 mA cm^{-2}. Scale bars in (a,b) are 20 μm.

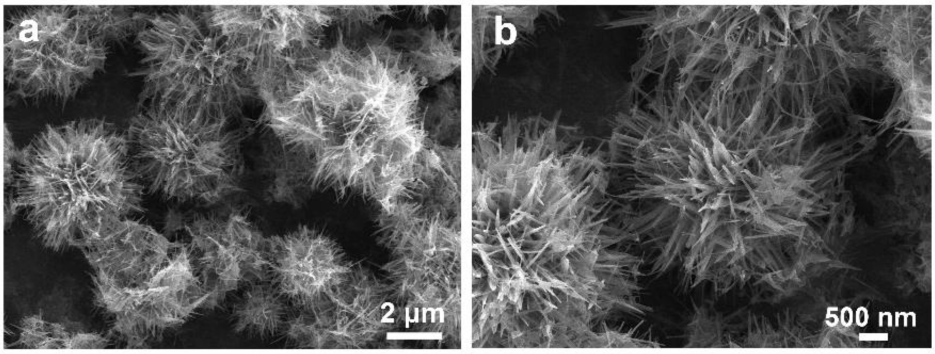

Figure S24. SEM images of the nanowire self-assembled MnO_2.

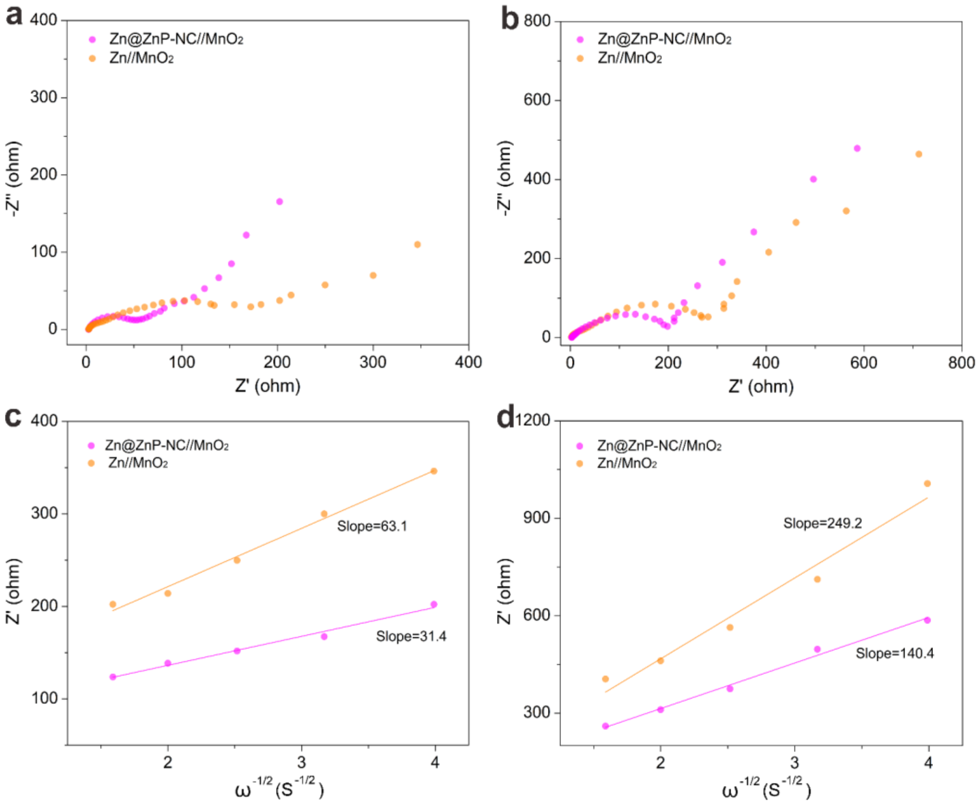

Figure S25. EIS plots of the Zn@ZnP-NC//MnO_2 and Zn//MnO_2 cells after (a) 10th cycle, and (b) 100th cycle. The corresponding Z' vs. in $\omega^{-1/2}$ curves in the low-frequency region after (c) 10th cycle and (d) 100 cycle.

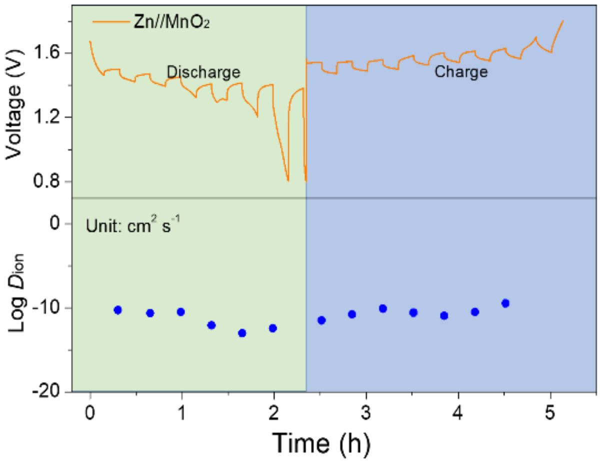

Figure S26. GITT profile and the corresponding diffusion coefficients of the Zn//MnO_2 full cell.

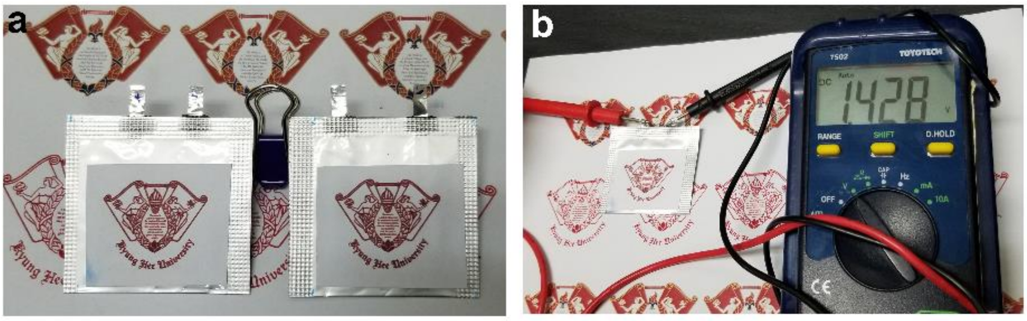

Figure S27. (a) Optical image and (b) corresponding open circuit voltage of the Zn@ZnP-NC//MnO_2 pouch cell.

The diffusion coefficient (D) can be calculated based on the following equation:

$$D = \frac{4}{\pi\tau}(\frac{m_B V_M}{M_B S})^2(\frac{\triangle E_S}{\triangle E_\tau})^2$$

where τ is the duration of the current pulse time, m_B. M_B, and V_M denote the active mass, molar mass, and molar volume of electrode material, respectively. S is the electrode-electrolyte interface area and ΔE_s is the quasi-thermodynamic equilibrium potential difference between before and after the current pulse regardless of the IR-drop. ΔE_τ represents the change of voltage during the current pulse.[16]

Reference

[1] a) J. Hao, B. Li, X. Li, X. Zeng, S. Zhang, F. Yang, S. Liu, D. Li, C. Wu, Z. Guo, *Adv. Mater.* **2020**, *32*, 2003021; b) M. Luo, C. Wang, H. Lu, Y. Lu, B. B. Xu, W. Sun, H. Pan, M. Yan, Y. Jiang, *Energy Stor. Mater.* **2021**, *41*, 515.

[2] Q. Cao, H. Gao, Y. Gao, J. Yang, C. Li, J. Pu, J. Du, J. Yang, D. Cai, Z. Pan, C. Guan, W. Huang, *Adv. Funct. Mater.* **2021**, *31*, 2103922.

[3] a) P. Zou, R. Zhang, L. Yao, J. Qin, K. Kisslinger, H. Zhuang, H. L. Xin, *Adv. Energy Mater.* **2021**, *11*, 2100982; b) S. Zhou, Y. Wang, H. Lu, Y. Zhang, C. Fu, I. Usman, Z. Liu, M. Feng, G. Fang, X. Cao, S. Liang, A. Pan, *Adv. Funct. Mater.* **2021**, *31*, 2104361.

[4] Q. Zhang, J. Luan, L. Fu, S. Wu, Y. Tang, X. Ji, H. Wang, *Angew. Chem., Int. Ed.* **2019**, *131*, 15988.

[5] Q. Lu, C. Liu, Y. Du, X. Wang, L. Ding, A. Omar, D. Mikhailova, *ACS Appl. Mater. Interfaces* **2021**, *13*, 16869.

[6] Q. Zhang, J. Luan, L. Fu, S. Wu, Y. Tang, X. Ji, H. Wang, *Nat. Commun.* **2020**, *11*, 3961.

[7] Y. Yang, C. Liu, Z. Lv, H. Yang, Y. Zhang, M. Ye, L. Chen, J. Zhao, C. C. Li, *Adv. Mater.* **2021**, *33*, 2007388.

[8] Y. Tian, Y. An, C. Wei, B. Xi, S. Xiong, J. Feng, Y. Qian, *ACS Nano* **2019**, *13*, 11676.

[9] Y. Yang, C. Liu, Z. Lv, H. Yang, X. Cheng, S. Zhang, M. Ye, Y. Zhang, L. Chen, J. Zhao, C. C. Li, *Energy Stor. Mater.* **2021**, *41*, 230.

[10] S. Li, J. Fu, G. Miao, S. Wang, W. Zhao, Z. Wu, Y. Zhang, X. Yang, *Adv. Mater.* **2021**, *33*, 2008424.

[11] Q. Cao, H. Gao, Y. Gao, J. Yang, C. Li, J. Pu, J. Du, J. Yang, D. Cai, Z. Pan, C. Guan, W. Huang, *Adv. Funct. Mater.* **2021**, *31*, 2103922.

[12] K. Hu, X. Guan, R. Lv, G. Li, Z. Hu, L. Ren, A. Wang, X. Liu, J. Luo, *Chem. Eng. J* **2020**, *396*, 125363.

[13] D. Han, S. Wu, S. Zhang, Y. Deng, C. Cui, L. Zhang, Y. Long, H. Li, Y. Tao, Z. Weng, Q. H. Yang, F. Kang, *Small* **2020**, *16*, 2001736.

[14] M. Zhou, S. Guo, J. Li, X. Luo, Z. Liu, T. Zhang, X. Cao, M. Long, B. Lu, A. Pan, G. Fang, J. Zhou, S. Liang, *Adv. Mater.* **2021**, *33*, 2100187.

[15] Z. Cao, X. Zhu, D. Xu, P. Dong, M. O. L. Chee, X. Li, K. Zhu, M. Ye, J. Shen, *Energy Stor. Mater.* **2021**, *36*, 132.

[16] a) Y. An, Y. Tian, S. Xiong, J. Feng, Y. Qian, *ACS Nano* **2021**, *15*, 11828; b) B. Wu, G. Zhang, M. Yan, T. Xiong, P. He, L. He, X. Xu, L. Mai, *Small* **2018**, *14*, 1703850.
